# Mycoplasma-associated multidrug resistance of hepatocarcinoma cells requires the interaction of P37 and Annexin A2

**DOI:** 10.1371/journal.pone.0184578

**Published:** 2017-10-04

**Authors:** Danyang Liu, Yang Hu, Ying Guo, Zhu Zhu, Bingzheng Lu, Xuelan Wang, Yijun Huang

**Affiliations:** 1 Department of Pharmacology, Zhongshan School of Medicine, Sun Yat-sen University, Guangzhou, China; 2 State Key Laboratory of Oncology in South China, Collaborative Innovation Centre for Cancer Medicine, Sun Yat-sen University Cancer Centre, Guangzhou, China; University of Navarra, SPAIN

## Abstract

Mycoplasma infection has been reported to be associated with cancer migration, invasion, epithelial-mesenchymal transition as well as the resistance to nucleoside analogues chemotherapeutic drugs. In this study, we found that the sensitivity of hepatocarcinoma cells to Cisplatin, Gemcitabine and Mitoxantrone was increased by mycoplasma elimination. Similar to the effect of anti-mycoplasma agent, interrupting the interaction between *Mycoplasma hyorhinis* membrane protein P37 and Annexin A2 of host cells using the N-terminal of ANXA2 polypeptide enhanced the sensitivity of HCC97L cells to Gemcitabine and Mitoxantrone. Meanwhile, we did not observe any changes in expression or distribution of multidrug resistance associated transporters, ATP-Binding Cassette protein B1, C1 and G2, on the removal of mycoplasma. These results suggest that mycoplasma induces a resistance to multiple drugs in hepatocarcinoma cells which required the interaction of P37 and Annexin A2. The pathway downstream this interaction needs to be explored.

## Introduction

Mycoplasma is a smallon prokaryotic microorganism found in man epithelial tissues[1] and body cavity such as urethra[2], alimentary canal[3] and respiratory tract[4]. Mycoplasma has also been detected in many kinds of human carcinomas such as lung cancer, gastric carcinoma, colon carcinoma[5], and hepatocellular carcinoma[6], with known influence mainly on tumor initiation, epithelial-mesenchymal transition, migration and invasion[7–9]. Recent works suggested that mycoplasma infection result in drug resistance to nucleoside analogues in cancer cells[10,11]. However, it remains unexplored whether mycoplasma has an effect on tumor cell sensitivity to a broader range of cytotoxic insults.

To date, mycoplasma is reported to affect host cells via their extracytoplasmic binding lipoproteins such as P37 of *Mycoplasma hyorhinis*[12]. Previous studies showed that P37 of *Mycoplasma hyorhinis* promoted migration of cancer cells by interacting with N-terminal of Annexin A2 (ANXA2)[13] which is an Annexin family protein existing in numerous kinds of cells[14] and associates with exocytosis, endocytosis and cell-cell adhesion[15]. On the other hand, the interaction between P37 and ANXA2 could be blocked by a 30 amino acid polypeptide within the N-terminal of ANXA2 (A2PP), leading to suppression of mycoplasma-induced migration and invasion[16]. These findings indicated the importance of P37-ANXA2 interaction in tumor progression. Meanwhile, ANXA2 had been found to be involved in the multidrug resistance (MDR) of tumor cells to chemotherapeutic agents including cisplatin, 5-fluorouracil, Doxorubicin and Topotecan[17,18]. If mycoplasma really works on the sensitivity to a wide variety of drugs in tumor cells, is the effect also initiated by the interaction of P37 and ANXA2?

MDR is a major contributor to the survival of cancer cells exposed to several drugs unrelated in both structure and mechanism[19–21]. Radio- and chemo- therapy themselves are well known inducers of cancer cell MDR, while the role of other environmental including biological factor(s) in MDR of cancers remains to be elucidated. Active efflux systems, especially ATP-Binding Cassette (ABC) transporter family members ABCB1 (P-gp/MDR1), ABCC1 (MRP1) and ABCG2 (BCRP/MXR/ABCP) play a critical role in MDR[22–24] by exclusion of hundreds of structurally diverse substrates[25] including endogenous metabolites, Glucuronide conjugates and GSH conjugates[26], and cytotoxic agents such as cisplatin, gemcitabine and mitoxantrone[27,28]. It is still unknown whether mycoplasma infection influences these universal effectors for MDR.

We here provide evidences that mycoplasma infection was involved in a resistance of hepatocarcinoma cells to chemotherapeutic drugs with different structures and mechanisms. We then observed the effect of blocking the interaction of P37 and ANXA2 on this resistance, and investigate its putative mechanism.

## Material and methods

### 1. Drugs and reagents

Cisplatin (CDDP) was purchased from Hospira Australia Pty Ltd. (Victoria, Australia). Gemcitabine Hydrochloride for Injection (GEM) was purchased from Eli Lilly and Company (Indiana, USA). Mitoxantrone Hydrochloride Injection (MX) was purchased from Sichuan Shenghe Pharmaceutical Co., Ltd. (Sichuan, China). Moxifloxacin Hydrochloride and Sodium Chloride Injection (MXF) were purchased from Bayer Ltd. (Leverkusen, Germany). Azithromycin for Injection (AZI) was purchased from Pfizer (Nk, USA). The primary antibodies were rabbit monoclonal ABCB1 antibody (Cell Signaling Technology, Sydney, Australia), rabbit monoclonal ABCC1 antibody (Cell Signaling Technology, Sydney, Australia), and mouse monoclonal ABCG2 antibody (Santa Cruz Biotechnology, Texas, USA). Mouse monoclonal β-actin antibody (Thermo Fisher Scientific, MA, USA) was used as an internal reference. Mouse monoclonal ZO-1 antibody (Thermo Fisher Scientific, MA, USA) and rabbit polyclonal ZO-1 antibody (Thermo Fisher Scientific, MA, USA) were used to delimitate the membrane in immunofluorescence (S1 Table). Secondary antibodies were horse anti-mouse/rabbit IgG-horseradish peroxidase (Cell Signaling Technology, Sydney, Australia) for Western blotting. Alexa Fluor-conjugated anti-rabbit and Alexa Fluor-conjugated anti-mouse secondary antibodies (Thermo Fisher Scientific, MA, USA) were used for immunoflourscence (S2 Table).

### 2. Cell culture

The human liver cancer cell line HCC97L was obtained from Zhongshan Hospital Affiliated to Fudan University (Shanghai, China). Hep3B and PLC/PRF/5 cell lines were obtained from American Type Culture Collection (ATCC, Manassas, USA). HCC97L and Hep3B were cultured in RPMI 1640 medium (Corning, NY, USA) while PLC/PRF/5 in DMEM (Corning, NY, USA) containing 10% fetal bovine serum (FBS, Biowest, Nuaillé, France), 100 U/mL penicillin, and 100 U/mL streptomycin (PAN-Biotech GmbH, Aidenbach Bavaria, Germany). All the cells were incubated at 37°C with 5% CO_2_ and 95% relative humidity.

### 3. Mycoplasma detection using quantitative real-time PCR

Total DNA was extracted from 5×10^5^ cells from each group by digestion at 70°C for 10 min in 0.5% Tween-20, 50 mM Tris (pH 8.5), 1 mM EDTA, and 200 mg/L proteinase K, followed by phenol/chloroform/isoamyl alcohol extraction and sodium acetate precipitation. DNA precipitates were washed with 70% ethanol, dried, and dissolved in 20 μL of sterile water. The extracted DNA (1μL, 1.2 μg) from the cells was added to 9 μL of the reaction solution, containing PCR buffer (SuperReal PreMix, SYBR Green; Tiangen Biotech, Beijing, China) and primers pairs for mycoplasma detection and reference control (β-actin) with a final concentration of 0.12 μM for each primer, to a total volume of 10 μL. Quantitative real-time PCR (qPCR) was performed as the following profile: 95°C for 1min (preincubation), followed by 40 cycles at 95°C for 10 s (denaturation), 60°C for 30 s (annealing and elongation). The primers (Table 1) for mycoplasma detection were gifts from Prof. Zhongning Lin (School of Public Health, Xiamen University) including two forward primers for universal mycoplasma detection, one forward primer to detect *Myco M*. *pirum*., one for *Myco A*. *laidlawii*. detection and a mix of degenerate primers to work as the reverse primers. All the primers were verified by NCBI Primer-Blast.

**Table 1:** Primers used for qPCR of mycoplasma detection.

Primers	Sequences
β-actin (F)	5’-GATCATTGCTCCTCCTGAGC-3’
β-actin (R)	5’-ACTCCTGCTTGCTGATCCAC-3’
Myco 6Mix.A (F)	5'-TCTGAATCTGCCGGGACCACC-3'
Myco 6Mix.B (F)	5'-TCTGAATTTGCCGGGACCACC-3'
*Myco M*. *pirum*. (F)	5'-GGAAAATGTTATTTTGACGGAACCT-3'
*Myco A*. *laidlawii*. (F)	5'-GGAATCCCGTTTGAAGATAGGA-3'
*Myco 8Mix*. (R)	5'-CTTTCC(A/C)TCAC(G/T)GTACT(A/G)GTTCACT-3'

F: forward primer; R: reverse primer.

### 4. Drug treatment and cytotoxicity assay

HCC97L and Hep3B cells were pretreated with 3 μg/mL (or 1μg/mL for Hep3B cell line) Moxifloxacin or 5 μg/mL Azithromycin respectively for 5 days. Then, HCC97L (3×10^3^ well^-1^) and Hep3B (4×10^3^ well^-1^) were seeded in 96-well plates (100 μL/well) and allowed to attach for 24 h. Then, each cell line was treated with 3 chemotherapeutic drugs separately at different concentrations in the presence of the anti-mycoplasma antibiotics for 48h. In the P37-ANXA2 interruption experiment, A2PP was dissolved in DMSO and HCC97L cells were pretreated with A2PP 24 h before GEM or MX treatment. 10 μL of MTT (5mg/ml final concentration; MP Biomedicals, LLC, CA, USA) was added to each well. After 4 h incubation at 37°C, the medium was removed and 100 μL/well DMSO (GBCBIO Technologies, Guangzhou, China) was added. The plates were mixed by a thermomixer for 5 min at RT. Absorbance measures were made in a microplate reader (iMark, Bio-Rad Laboratories, CA, USA) at 570/655nm.

### 5. Western blot

Cells were washed by ice-cold PBS and lysed in Tris-NaCl buffer (50 mM Tris pH 7.4, 150mM NaCl, 25 mM EDTA, 1 mM NaF, Protease Inhibitor Cocktail, 1 mM PMSF and 1% Triton X-100) on ice for 20 min. Then the lysate was centrifuged at 12000 rmp for 15 min at 4°C.Protein concentration was determined using Thermo Scientific Pierce BCA protein assay kit (Pierce, Rockford, IL, USA) according the manufacturer’s recommendation. For protein separation, equal amounts of protein (30 μg) were separated by 8% SDS-polyacrylamide gel electrophoresis and transferred onto polyvinylidene difluoride membranes (Millipore Corporation, MA, USA). The blots were blocked in 5% skim milk for 1 h at RT and incubated overnight with primary antibodies at 4°C, followed by incubating with secondary antibodies for 1h at RT. The blots were washed three times with TBS-T, each for 5 min, and incubated with Western Lightning Chemiluminescence Reagent Plus ECL kit (Amersham, USA) for 1 min to measure the protein expression. Protein band densitometry was performed using ImageJ software (National Institutes of Health).

### 6. Immunoflourscence staining

Cells were cultured and treated directly in chamber slides and were fixed with 4% formaldehyde for 15 min at RT. After rinsing slides three times in PBS for 5 min each, cells were blocked in 5% BSA for 1 h. Primary antibodies were applied to the cells overnight at 4°C, followed by fluorochrome-conjugated secondary antibodies and Hoechst33342 1–2 h at room temperature in the dark. A Nikon A1 confocal system (Nikon, Tokyo, Japan) was used to observe the location of indicated proteins. Digital images were arranged by Adobe Photoshop CS4 (Adobe Systems).

### 7. Statistical analysis

All the experiments were performed in triplicate. All data are presented as mean ± SD. Paired two-tailed student’s *t*-test was used for comparison between two groups, extra-sum-of-squares *F* tests was used for dose-response curves, and all performed using GraphPad PRISM 5 (GraphPad Software, San Diego, California, USA). The significance level was set at *P* < 0.05. No randomization or blinding was used in the studies.

## Data availability statement

The data that support the findings of this study are available from the corresponding author upon reasonable request.

## Results

### 1. Moxifloxacin and Azithromycin eliminated mycoplasma in vitro

**Fig 1:**
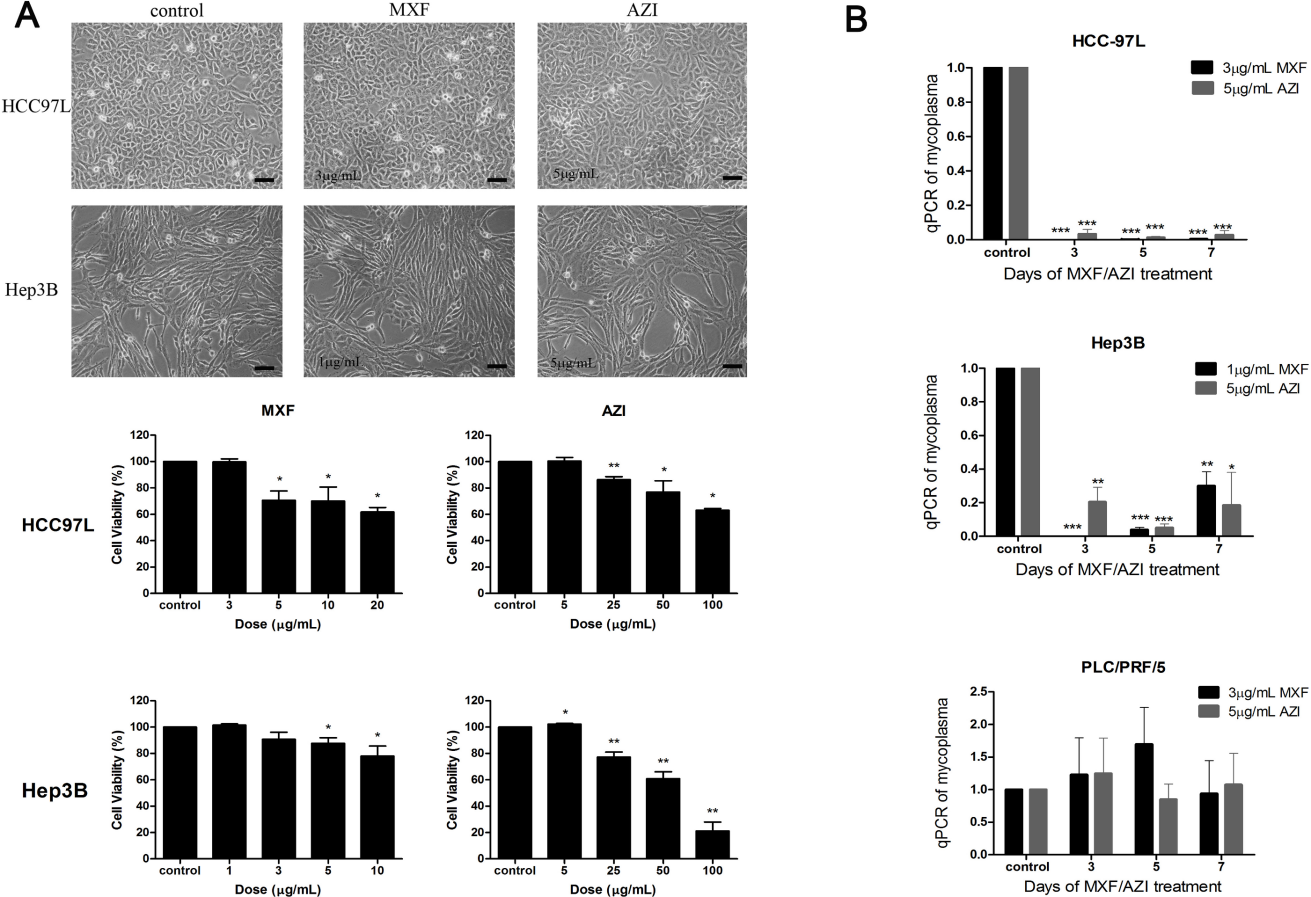
The cytotoxic and anti-mycoplasma effect of anti-mycoplasma antibiotics on hepatocarcinoma cells. (A) Images showed cytotoxic effect of AZI/MXF on HCC97L/Hep3B cells, respectively. (×200; bar, 50 μm); MTT analysis showed the cell viability of HCC97L/Hep3B treated with increasing concentrations of AZI/MXF, respectively. (B) The relative mycoplasma DNA copy numbers measured using qPCR showed the anti-mycoplasma effect of AZI/MXF treatment on HCC97L, Hep3B and PLC/PRF/5 cells for 3, 5 or 7 days. Error bars indicate SD of a representative experiment out of three independent experiments performed in triplicate. Statistical significance was determined by using paired two-tailed student’s *t*-test: ***P < 0.001, ***P < 0.01, *P < 0.05 as compared with control. *P* values, *t*-values and degree of freedom were provided in S3 and S4 Tables.

In this study, we treated cells with two antibiotics: a macrolide agent, Azithromycin (AZI), and a fluoroquinolone agent, Moxifloxacin (MXF). Cell morphology and MTT assay showed that 3 μg/mL MXF and 5 μg/mL AZI were non-toxic to HCC97L cells as well as 1 μg/mL MXF and 5 μg/mL AZI to Hep3B (Fig 1A). qPCR analysis indicated that 1 μg/mL and 3 μg/mL MXF eradicated mycoplasma completely in Hep3B and HCC97L cells respectively, while 5 μg/mL AZI removed mycoplasma significantly in both cell lines. In PLC/PRF/5 cell line, however, mycoplasma remained intact under the treatment of 3 μg/mL MXF or 5 μg/mL AZI (Fig 1B).

### 2. Moxifloxacin and Azithromycin enhanced the sensitivity of human hepatocellular carcinoma cell lines to chemotherapeutic drugs

**Fig 2:**
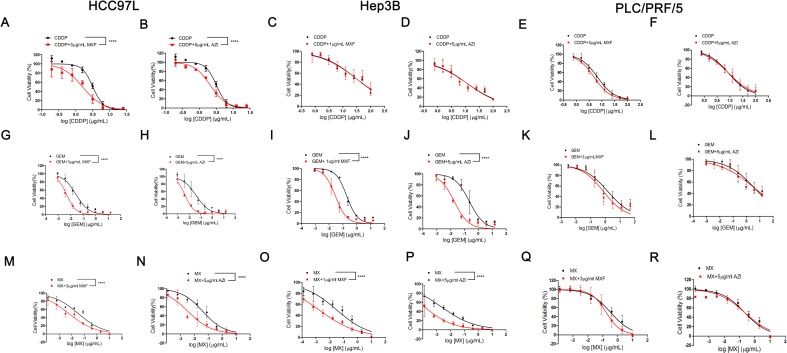
The cell viability of hepatocarcinoma cells treated with different chemotheraputic drugs alone or with the presence of anti-mycoplasma antibiotics. Cell viability of HCC97L (A/G/M and B/H/N), Hep3B (C/I/O and D/J/P) and PLC/PRF/5 cell (E/K/Q and F/L/R), which were treated with CDDP/GEM/MX with or without AZI/MXF at the indicated concentrations. Error bars indicate SD of a representative experiment out of three independent experiments performed in triplicate. Statistical testing was performed by comparing the logIC_50_ values by means of an extra-sum-of-squares *F* test. ****P < 0.0001 as compared to the chemotherapeutic drug alone controls. IC_50_ values, *F* values, degrees of freedom (DFn, DFd) and *P* values were provided in S5 Table.

We then treated cells with an alkylating agent, CDDP, an antimetabolite, GEM, and an anthracycline topoisomerase inhibitor, MX, respectively with or without the existence of non-cytotoxic concentration of MXF or AZI. The results of MTT assay indicated that the sensitivity of HCC97L cells to CDDP, GEM and MX, and the sensitivity of Hep3B cells to GEM and MX were enhanced by MXF and AZI, while CDDP cytotoxicity against Hep3B cells did not benefit from combination with MXF or AZI. In PLC/PRF/5 cell line, neither MXF nor AZI improved the efficacy of the anti-tumor drugs (Fig 2).

### 3. Mycoplasma-related MDR required the interaction of P37 and Annexin A2

**Fig 3:**
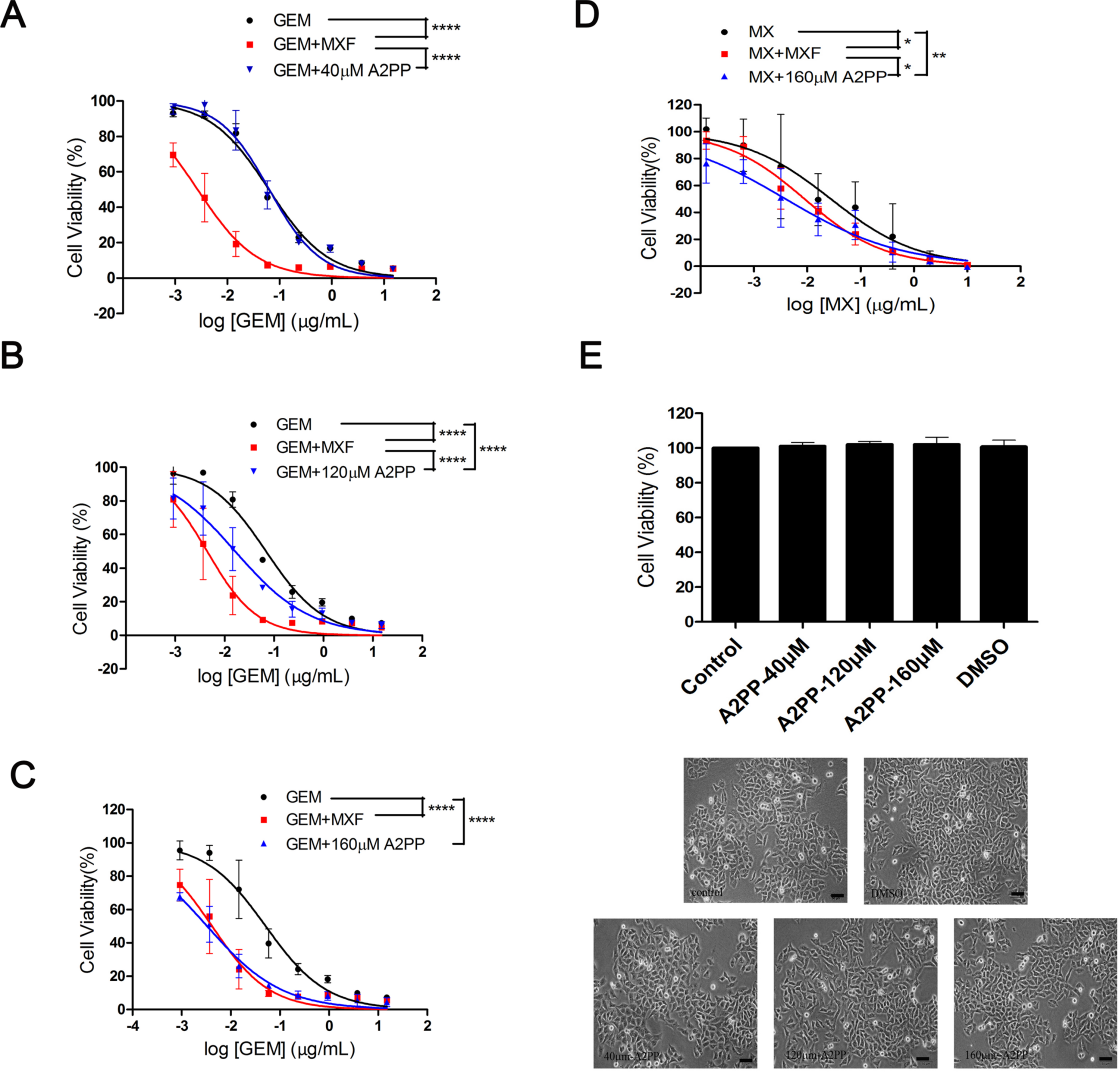
The effect of A2PP/MXF on the sensitivity of HCC97L to chemotherapeutic drugs. (A, B and C) Cell viability of HCC97L cells treated using GEM alone, GEM with the presence 3μg/mL of MXF or GEM with with three different concentrations of A2PP, which were 40 μM, 120 μM and 160 μM respectively. (D) Cell viability of HCC97L cells treated using MX alone, MX with the presence of MXF or MX with 160 μM of A2PP. Statistical testing was performed by comparing the logIC_50_ values by means of an extra-sum-of-squares *F* test. ****P < 0.0001, ***P < 0.001, **P < 0.01, *P < 0.05 as compared with each group. IC_50_ values, *F* values, degrees of freedom (DFn, DFd) and P values were provided in S6 Table. (E) Cell viability of HCC97L cells treated with different concentrations of A2PP or vehicle DMSO for 72 h (×200; bar, 50 μm). Statistical significance was determined by using paired two-tailed student’s *t*-test. *P* values, *t*-values and degree of freedom were provided in S7 Table. Error bars indicate SD of a representative experiment out of three independent experiments performed in triplicate.

To explore the initiation of infection-related MDR, we employed A2PP to block the P37 protein from binding ANXA2. With the presence of the increasing concentration of A2PP, GEM exerted stronger inhibiting effect than treatment alone in HCC97L cell line. Impressively, the maximum effect of GEM with A2PP was equivalent to that with MXF (Fig 3A, 3B and 3C). This enhancement by A2PP was reproduced when used together with MX (Fig 3D). In addition, cell morphology and MTT assay showed that A2PP had no impact on cell survival (Fig 3E), indicating that the augmentation of the anti-tumor effect by A2PP could not result from its direct cytotoxicity.

### 4. ABC transporters were not involved in mycoplasma-related MDR

**Fig 4:**
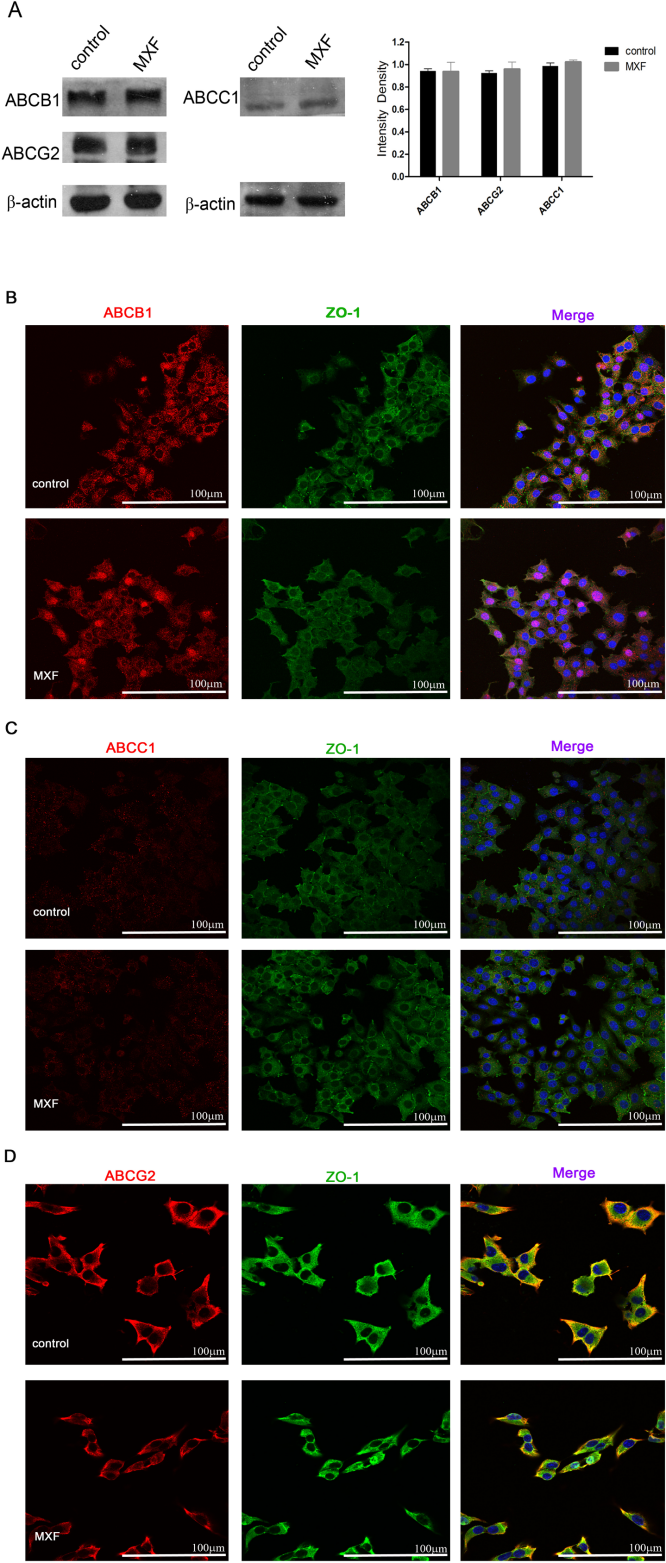
The expression and subcellular location changes of ABC transporter family proteins with or without MXF treatment. (A) Protein expressions of ABCB1, ABCC1 and ABCG2 in HCC-97L cells treated with MXF for 7 days compared with non-treated controls. Statistical significance was determined by using unpaired two-tailed student’s *t*-test. *P* values, *t*-values and degrees of freedom were provided in S8 Table. Error bars indicate SD of a representative experiment out of three independent experiments performed in triplicate. The subcellular locations of ABCB1 (B), ABC1 (C) and ABCG2 (D) in HCC-97L cells treated with MXF for 7 days or non-treated controls. ZO-1 was used to delimitate the membrane (×400; bar, 100 μm).

To figure out the cellular effector responsible for the mycoplasma-related MDR, we measured the expression and sub-cellular location of three ABC transporter members: ABCB1, ABCC1 and ABCG2. Interestingly, no substantial change in protein quantity of these transporters was observed with MXF treatment. The distribution of these proteins on cell membrane did not alter either. (Fig 4).

## Discussion

Although interest and fascination around the relationship between microorganism and human body have been reignited by some amazing findings which exhibited the complicated and surprising patterns how bacteria affect physiological functions and pathogenesis of human body[29–32], much more work needs to be done to understand how human-bacteria interact. As a class of microorganism, mycoplasma has been revealed associated to many human diseases, as notorious as *M*. *genitalium* in sexually transmitted disease syndromes[33] and *M*. *pneumoniae* in community acquired pneumonia (CAP)[34]. Since the relationship between mycoplasma and tumor was first reported in human leukemia and lymphoma decades ago[35], mycoplasma has been found important to progression of an increasing variety of tumors[5] in different ways[36–39]. Given the fact that tumor cells always encounter tough challenges such as intracellular accumulation of reactive oxygen species (ROS) due to rapid metabolism[40,41], and severe damage by chemotherapy and radiotherapy, it is reasonable to speculate that mycoplasma would not stand aside watching its feeding cells suffer and die. We propose that mycobacteria have a role in the response to these cytotoxic stimuli.

In this study, all cell lines were cultured according to standard procedures: no special treatment to prevent mycoplasma infection and cells were not deliberately exposed to the microorganism Such protocol led to 100% mycoplasma contamination rates, mimicking the natural situation that tissue cells are susceptible to mycoplasma infection. During the experiments, we employed two antibiotics with totally different mechanisms to contain mycoplasma: MXF, a fluoroquinolones which inhibits topoisomerase, and AZI, a macrolides which targets ribosome for protein synthesis in bacterium. To identify the real origin of the enhanced cytotoxicity from anti-tumor treatment combined with MXF or AZI, evidences were pooled together for analysis: (1) MXF and AZI were introduced at non-cytotoxic concentrations; (2) in cell lines where mycoplasma was cleaned up by MXF and AZI, cytotoxicity of anti-tumor drugs was intensified; (3) in cell line where mycoplasma survived MXF and AZI, cytotoxicity of anti-tumor drugs remained unchanged. It can be reasoned out that the augmentation of the anti-tumor activity on each combination resulted from the elimination of mycoplasma rather than the direct cytotoxicity of MXF or AZI. That means the presence of mycoplasma is the driving factor of a MDR in those tumor cells.

*Mycoplasma hyorhinis* membrane protein P37 is a functional protein intensively studied for its role in tumor behaviors[36,42]. ANXA2[43] and HER2[44] are two known binding sites of P37. Recent data indicated that P37 promoted tumor progression through its interaction with ANXA2 in host cells, while P37 antibodies, a polypeptide A2PP (a 30 amino acids polypeptide within the N-terminal of ANXA2), and anti-mycoplasma reagent like MYCO I, were able to block this interaction[16]. We demonstrated here that non-cytotoxic levels of A2PP improved the sensitivity of tumor cells to chemotherapeutic drugs, very similar to the effect of mycoplasma removal. This result strongly supports the previous deduction that mycoplasma induced a MDR of tumor cells, and indicates the interaction of P37 and ANXA2 the initial step of this MDR.

Previously, Bronckaers *et al* revealed that pyrimidine nucleoside analogues could be degraded in mycoplasma infected tumor cells in 2008[45]. In *Mycoplasma hyorhinis*-infected tumor cell cultures, mycoplasma-encoded cytidine deaminase (CDA) and pyrimidine nucleoside phosphorylase (PyNP) compromise the antitumor activity of nucleoside analogues such as gemcitabine and 5-FU by deamination [38,39]. Given the irrelevant structures and mechanisms of CDDP, MX and GEM, specific degradation of nucleoside analogues by CDA/PyNP cannot explain the mycoplasma-related MDR in this study, implying a universal effector for this phenomenon. Considering the ability of ABC transporters to exclude a broad range of substrates, we tested 3 ABC transporters. However, we did not detect any changes in expression or sub-cellular locations of ABCB1, ABCC1 and ABCG2, suggesting that mycoplasma infection alters neither the quantity of ABC transporters nor their functional proportions on cell membrane. Based on evidences in this study, admittedly, we cannot deny the possibility of other efflux transporter which executes mycoplasma-related MDR. Interestingly, a gene analysis in 1988 confirmed the homogeneity between P37 and ABC transporters[46,47], implying that P37 may play an important role in resistance of mycoplasma to antibiotics. We propose that P37 is recruited to tumor cell membrane by ANXA2 as exogenous transporter to pump out cytotoxic substrates for the survival of host cells as well as of themselves.

In conclusion, we found that mycoplasma infection gives rise to a MDR of human hepatocarcinoma cells and proved that this influence depends on the interaction between P37 of mycoplasma and Annexin A2 of host cells. These primary data may open a window for our extended understanding about the interaction between microorganism and human body including the abnormal parts. More work needed to determine the mechanism and downstream pathways involved in this observation. The spectrum of tumor cells whose defense against chemotherapeutic agents could be strengthened by mycoplasma should be known before the clinical meaning of anti-mycoplasma strategy in cancer management is pursued to greater depths.

## Supporting information

S1 Table The information of primary antibodies for western blotting and immunoflourscence staining. A list of all the primary antibodies for western blotting and immunoflourscence staining we used in this study.
(DOCX)

S2 Table The information of secondary antibodies for western blotting and immunoflourscence staining. A list of all the secondary antibodies for western blotting and immunoflourscence staining we used in this study.
(DOCX)

S3 Table The statistical data of paired two-tailed student’s t-test in Fig 1. **A.** P values, t values and degree of freedom in Fig 1A, the MTT analysis the cell viability of HCC97L/Hep3B treated with increasing concentrations of AZI/MXF, were analyzed using paired two-tailed student’s *t*-test.
(DOCX)

S4 Table The statistical data of paired two-tailed student’s t-test in Fig 1B. P values, t values and degree of freedom in Fig 1B, the qPCR analysis for the anti-mycoplasma effect of AZI/MXF treatment on HCC97L, Hep3B and PLC/PRF/5 cells, were analyzed using paired two-tailed student’s *t*-test.
(DOCX)

S5 Table The statistical data of extra-sum-of-squares F tests in Fig 2. IC_50_ values, F values, degrees of freedom (DFn, DFd) and P values of each curve in Fig 2, the MTT analysis for the cell viability of hepatocarcinoma cells treated with different chemotheraputic drugs alone or with the presence of anti-pcytoplasma antibiotics, were analyzed using extra-sum-of-squares F test.
(DOCX)

S6 Table The statistical data of extra-sum-of-squares F tests in Fig 3A–3D. IC_50_ values, F values, degrees of freedom (DFn, DFd) and P values of each curve in Fig 3A–3D, the MTT analysis for the effect of A2PP on the cell viability of HCC97L to chemotherapeutic drugs, were analyzed using extra-sum-of-squares F test.
(DOCX)

S7 Table The statistical data of paired two-tailed student’s t-test in Fig 3E. P values, t values and degree of freedom in Fig 3E, MTT analysis for Cell viability of HCC97L cells treated with different concentrations of A2PP or vehicle DMSO, were analyzed using paired two-tailed student’s *t*-test.
(DOCX)

S8 Table The statistical data of unpaired two-tailed student’s t-test in Fig 4A. P values, t values and degree of freedom in Fig 4A, the intensity density analysis for western blotting, were analyzed using unpaired two-tailed student’s *t*-test.
(DOCX)

S1 Fig The expression changes of ABC transporter family proteins with or without MXF treatment. (A) Full-length blots in Fig 4 are presented. (B) Multiple exposures of ABCB1, ABCC1 and ABCG2 are presented.
(TIF)

## References

[1] Razin S, Yogev D, Naot Y. Molecular biology and pathogenicity of mycoplasmas. Microbiol Mol Biol Rev. 1998; 62: 1094–1156. 9841667 10.1128/mmbr.62.4.1094-1156.1998 PMC98941

[2] Manhart LE, Jensen JS, Bradshaw CS, Golden MR, Martin DH. Efficacy of Antimicrobial Therapy for Mycoplasma genitalium Infections. Clin Infect Dis. 2015; 61 Suppl 8: S802–S817. 26602619 10.1093/cid/civ785

[3] Liekens S, Bronckaers A, Balzarini J. Improvement of purine and pyrimidine antimetabolite-based anticancer treatment by selective suppression of mycoplasma-encoded catabolic enzymes. Lancet Oncol. 2009; 10: 628–635. doi: 10.1016/S1470-2045(09)70037-3 19482252 10.1016/S1470-2045(09)70037-3

[4] Medjo B, Atanaskovic-Markovic M, Radic S, Nikolic D, Lukac M, Djukic S. Mycoplasma pneumoniae as a causative agent of community-acquired pneumonia in children: clinical features and laboratory diagnosis. Ital J Pediatr. 2014; 40: 104 doi: 10.1186/s13052-014-0104-4 25518734 10.1186/s13052-014-0104-4 PMC4279889

[5] Huang S, Li JY, Wu J, Meng L, Shou CC. Mycoplasma infections and different human carcinomas. World journal of gastroenterology: WJG. 2001; 7: 266 doi: 10.3748/wjg.v7.i2.266 11819772 10.3748/wjg.v7.i2.266 PMC4723534

[6] Choi HS, Lee HM, Kim W, Kim MK, Chang HJ, Lee HR, et al Detection of mycoplasma infection in circulating tumor cells in patients with hepatocellular carcinoma. Biochemical and Biophysical Research Communications. 2014; 446: 620–625. doi: 10.1016/j.bbrc.2014.03.024 24637212 10.1016/j.bbrc.2014.03.024

[7] Gong M, Meng L, Jiang B, Zhang J, Yang H, Wu J, et al p37 from Mycoplasma hyorhinis promotes cancer cell invasiveness and metastasis through activation of MMP-2 and followed by phosphorylation of EGFR. Mol Cancer Ther. 2008; 7: 530–537. doi: 10.1158/1535-7163.MCT-07-2191 18347140 10.1158/1535-7163.MCT-07-2191

[8] Ketcham CM, Anai S, Reutzel R, Sheng S, Schuster SM, Brenes RB, et al p37 Induces tumor invasiveness. Mol Cancer Ther. 2005; 4: 1031–1038. doi: 10.1158/1535-7163.MCT-05-0040 16020660 10.1158/1535-7163.MCT-05-0040

[9] Duan H, Qu L, Shou C. Mycoplasma hyorhinis induces epithelial-mesenchymal transition in gastric cancer cell MGC803 via TLR4-NF-κB signaling. Cancer Letters. 2014; 354: 447–454. doi: 10.1016/j.canlet.2014.08.018 25149064 10.1016/j.canlet.2014.08.018

[10] Vande VJ, Liekens S, Gago F, Balzarini J. The pyrimidine nucleoside phosphorylase of Mycoplasma hyorhinis and how it may affect nucleoside-based therapy. Nucleosides Nucleotides Nucleic Acids. 2014; 33: 394–402. doi: 10.1080/15257770.2013.851394 24940697 10.1080/15257770.2013.851394

[11] Vande VJ, Balzarini J, Liekens S. Mycoplasmas and cancer: focus on nucleoside metabolism. EXCLI J. 2014; 13: 300–322. 26417262 PMC4464442

[12] Sippel KH, Robbins AH, Reutzel R, Boehlein SK, Namiki K, Goodison S, et al Structural Insights into the Extracytoplasmic Thiamine-Binding Lipoprotein p37 of Mycoplasma hyorhinis. Journal of Bacteriology. 2009; 191: 2585–2592. doi: 10.1128/JB.01680-08 19233924 10.1128/JB.01680-08 PMC2668404

[13] Duan H, Chen L, Qu L, Yang H, Song SW, Han Y, et al Mycoplasma Hyorhinis Infection Promotes NF- B-Dependent Migration of Gastric Cancer Cells. Cancer Research. 2014; 74: 5782–5794. doi: 10.1158/0008-5472.CAN-14-0650 25136068 10.1158/0008-5472.CAN-14-0650

[14] Wang C, Lin C. Annexin A2: Its Molecular Regulation and Cellular Expression in Cancer Development. Disease Markers. 2014; 2014: 1–10. 10.1155/2014/308976 PMC3925611 24591759

[15] Waisman DM. Annexin II tetramer: structure and function. Mol Cell Biochem. 1995; 149–150: 301–322. 8569746 10.1007/BF01076592

[16] Yuan S, Qu L, Shou C. N-Terminal Polypeptide of Annexin A2 Decreases Infection of Mycoplasma hyorhinis to Gastric Cancer Cells. PLOS ONE. 2016; 11: e147776. 10.1371/journal.pone.0147776 PMC4727897 26812398

[17] ZHANG ZD, LI Y, FAN LQ, ZHAO Q, TAN BB, ZHAO XF. Annexin A2 is implicated in multi-drug-resistance in gastric cancer through p38MAPK and AKT pathway. Neoplasma. 2014; 61: 627–637. doi: 10.4149/neo_2014_078 25150310 10.4149/neo_2014_078

[18] Chen CY, Lin YS, Chen CL, Chao PZ, Chiou JF, Kuo CC, et al Targeting annexin A2 reduces tumorigenesis and therapeutic resistance of nasopharyngeal carcinoma. Oncotarget. 2015; 6: 26946–26959. doi: 10.18632/oncotarget.4521 26196246 10.18632/oncotarget.4521 PMC4694965

[19] Gottesman MM, Fojo T, Bates SE. MULTIDRUG RESISTANCE IN CANCER: ROLE OF ATP-DEPENDENT TRANSPORTERS. Nature Reviews Cancer. 2002; 2: 48–58. doi: 10.1038/nrc706 11902585 10.1038/nrc706

[20] Higgins CF. Multiple molecular mechanisms for multidrug resistance transporters. Nature. 2007; 446: 749–757. doi: 10.1038/nature05630 17429392 10.1038/nature05630

[21] Joyce H, McCann A, Clynes M, Larkin A. Influence of multidrug resistance and drug transport proteins on chemotherapy drug metabolism. Expert Opin Drug Metab Toxicol. 2015; 11: 795–809. doi: 10.1517/17425255.2015.1028356 25836015 10.1517/17425255.2015.1028356

[22] Dean M, Hamon Y, Chimini G. The human ATP-binding cassette (ABC) transporter superfamily. J Lipid Res. 2001; 42: 1007–1017. 11441126

[23] Sodani K, Patel A, Anreddy N, Singh S, Yang D, Kathawala RJ, et al Telatinib reverses chemotherapeutic multidrug resistance mediated by ABCG2 efflux transporter in vitro and in vivo. Biochemical Pharmacology. 2014; 89: 52–61. doi: 10.1016/j.bcp.2014.02.012 24565910 10.1016/j.bcp.2014.02.012 PMC3983711

[24] Saier MJ, Paulsen IT. Phylogeny of multidrug transporters. Semin Cell Dev Biol. 2001; 12: 205–213. doi: 10.1006/scdb.2000.0246 11428913 10.1006/scdb.2000.0246

[25] Eckford PDW, Sharom FJ. ABC Efflux Pump-Based Resistance to Chemotherapy Drugs. Chemical Reviews. 2009; 109: 2989–3011. doi: 10.1021/cr9000226 19583429 10.1021/cr9000226

[26] Haimeur A, Conseil G, Deeley RG, Cole SP. The MRP-related and BCRP/ABCG2 multidrug resistance proteins: biology, substrate specificity and regulation. Curr Drug Metab. 2004; 5: 21–53. 14965249 10.2174/1389200043489199

[27] Szakács G, Paterson JK, Ludwig JA, Booth-Genthe C, Gottesman MM. Targeting multidrug resistance in cancer. Nature Reviews Drug Discovery. 2006; 5: 219–234. doi: 10.1038/nrd1984 16518375 10.1038/nrd1984

[28] Deeley RG. Transmembrane Transport of Endo- and Xenobiotics by Mammalian ATP-Binding Cassette Multidrug Resistance Proteins. Physiological Reviews. 2006; 86: 849–899. doi: 10.1152/physrev.00035.2005 16816140 10.1152/physrev.00035.2005

[29] Wen L, Ley RE, Volchkov PY, Stranges PB, Avanesyan L, Stonebraker AC, et al Innate immunity and intestinal microbiota in the development of Type 1 diabetes. Nature. 2008; 455: 1109–1113. doi: 10.1038/nature07336 18806780 10.1038/nature07336 PMC2574766

[30] Kostic AD, Chun E, Robertson L, Glickman JN, Gallini CA, Michaud M, et al Fusobacterium nucleatum potentiates intestinal tumorigenesis and modulates the tumor-immune microenvironment. Cell Host Microbe. 2013; 14: 207–215. doi: 10.1016/j.chom.2013.07.007 23954159 10.1016/j.chom.2013.07.007 PMC3772512

[31] Rosenbaum M, Knight R, Leibel RL. The gut microbiota in human energy homeostasis and obesity. Trends Endocrinol Metab. 2015; 26: 493–501. doi: 10.1016/j.tem.2015.07.002 26257300 10.1016/j.tem.2015.07.002 PMC4862197

[32] Li J, Zhao F, Wang Y, Chen J, Tao J, Tian G, et al Gut microbiota dysbiosis contributes to the development of hypertension. Microbiome. 2017; 5: 14 doi: 10.1186/s40168-016-0222-x 28143587 10.1186/s40168-016-0222-x PMC5286796

[33] Manhart LE. Mycoplasma genitalium: An emergent sexually transmitted disease? Infect Dis Clin North Am. 2013; 27: 779–792. doi: 10.1016/j.idc.2013.08.003 24275270 10.1016/j.idc.2013.08.003

[34] Chaudhry R, Ghosh A, Chandolia A. Pathogenesis of Mycoplasma pneumoniae: An update. Indian J Med Microbiol. 2016; 34: 7–16. doi: 10.4103/0255-0857.174112 26776112 10.4103/0255-0857.174112

[35] Murphy WH, Bullis C, Ertel IJ, Zarafonetis CJ. Mycoplasma studies of human leukemia. Ann N Y Acad Sci. 1967; 143: 544–556. 5233787 10.1111/j.1749-6632.1967.tb27701.x

[36] Steinemann C, Fenner M, Binz H, Parish RW. Invasive behavior of mouse sarcoma cells is inhibited by blocking a 37,000-dalton plasma membrane glycoprotein with Fab fragments. Proc Natl Acad Sci U S A. 1984; 81: 3747–3750. 6587388 10.1073/pnas.81.12.3747 PMC345296

[37] Vande Voorde J, Liekens S, Balzarini J. Mycoplasma hyorhinis-Encoded Purine Nucleoside Phosphorylase: Kinetic Properties and Its Effect on the Cytostatic Potential of Purine-Based Anticancer Drugs. Molecular Pharmacology. 2013; 84: 865–875. doi: 10.1124/mol.113.088625 24068428 10.1124/mol.113.088625

[38] Vande Voorde J, Sabuncuo Lu S, Noppen S, Hofer A, Ranjbarian F, Fieuws S, et al Nucleoside-catabolizing Enzymes in Mycoplasma-infected Tumor Cell Cultures Compromise the Cytostatic Activity of the Anticancer Drug Gemcitabine. Journal of Biological Chemistry. 2014; 289: 13054–13065. doi: 10.1074/jbc.M114.558924 24668817 10.1074/jbc.M114.558924 PMC4036319

[39] Vande Voorde J, Vervaeke P, Liekens S, Balzarini J. Mycoplasma hyorhinis-encoded cytidine deaminase efficiently inactivates cytosine-based anticancer drugs. FEBS Open Bio. 2015; 5: 634–639. doi: 10.1016/j.fob.2015.07.007 26322268 10.1016/j.fob.2015.07.007 PMC4541722

[40] Brenner C, Grimm S. The permeability transition pore complex in cancer cell death. Oncogene. 2006; 25: 4744–4756. doi: 10.1038/sj.onc.1209609 16892087 10.1038/sj.onc.1209609

[41] Wang J, Yi J. Cancer cell killing via ROS: to increase or decrease, that is the question. Cancer Biol Ther. 2008; 7: 1875–1884. 18981733 10.4161/cbt.7.12.7067

[42] Steinemann C, Fenner M, Parish RW, Binz H. Studies of the invasiveness of the chemically induced mouse sarcoma FS9. I. Monoclonal antibodies to a 37,000 dalton membrane glycoprotein inhibit invasion of fibroblasts in vitro. Int J Cancer. 1984; 34: 407–414. 6384068 10.1002/ijc.2910340319

[43] Duan H, Chen L, Qu L, Yang H, Song SW, Han Y, et al Mycoplasma Hyorhinis Infection Promotes NF- B-Dependent Migration of Gastric Cancer Cells. Cancer Research. 2014; 74: 5782–5794. doi: 10.1158/0008-5472.CAN-14-0650 25136068 10.1158/0008-5472.CAN-14-0650

[44] Wu J, Wu L, Fang C, Nie R, Wang J, Wang X, et al Mycoplasmal lipoprotein p37 binds human protein HER2. Microbiol Res. 2016; 192: 253–259. doi: 10.1016/j.micres.2016.08.003 27664744 10.1016/j.micres.2016.08.003

[45] Bronckaers A, Balzarini J, Liekens S. The cytostatic activity of pyrimidine nucleosides is strongly modulated by Mycoplasma hyorhinis infection: Implications for cancer therapy. Biochem Pharmacol. 2008; 76: 188–197. doi: 10.1016/j.bcp.2008.04.019 18555978 10.1016/j.bcp.2008.04.019

[46] Raherison S, Gonzalez P, Renaudin H, Charron A, Bebear C, Bebear CM. Increased expression of two multidrug transporter-like genes is associated with ethidium bromide and ciprofloxacin resistance in Mycoplasma hominis. Antimicrob Agents Chemother. 2005; 49: 421–424. doi: 10.1128/AAC.49.1.421-424.2005 15616325 10.1128/AAC.49.1.421-424.2005 PMC538859

[47] Dudler R, Schmidhauser C, Parish RW, Wettenhall RE, Schmidt T. A mycoplasma high-affinity transport system and the in vitro invasiveness of mouse sarcoma cells. EMBO J. 1988; 7: 3963–3970. 3208756 10.1002/j.1460-2075.1988.tb03283.x PMC454995

