# Supplementary material for: Mycoplasma-associated multidrug resistance of hepatocarcinoma cells requires the interaction of P37 and Annexin A2
Source: PLoS One. 2017 Oct 4;12(10):e0184578. doi: 10.1371/journal.pone.0184578 (PMC5627893; doi:10.1371/journal.pone.0184578)
Supplement: S1 Table — A list of all the primary antibodies for western blotting and immunoflourscence staining we used in this study. (DOCX) [file pone.0184578.s001.docx]

S1Table. The Information of Primary Antibodies for Western blotting and Immunoflourscence staining

| Catalog number | Name | Provider | Dilutions |
| --- | --- | --- | --- |
| 13342 | MDR1/ABCB1 (E1Y7B) Rabbit mAb | Cell Signaling Technology, Inc | Western blotting 1:1000  IF-IC 1:800 |
| 14685 | MRP1/ABCC1 (D7O8N) Rabbit mAb | Cell Signaling Technology, Inc | Western blotting 1:1000  IF-IC 1:200 |
| sc-377176 | ABCG2(B-1) mouse mAb | Santa Cruz Biotechnology, Inc | Western blotting 1:100  IF-IC 1:200 |
| MA5-15739 | Anti-beta Actin Loading Control Monoclonal Antibody (BA3R) | Invitrogen Antibodies | Western blotting 1:1000 |
| 61-7300 | Anti-ZO-1 Polyclonal Antibody | Invitrogen Antibodies | IF-IC 1:50 |
| 33-9111 | Anti-ZO-1 Monoclonal Antibody (ZO1-1A12), FITC | Invitrogen Antibodies | IF-IC 1:100 |
