# Supplementary material for: Mycoplasma-associated multidrug resistance of hepatocarcinoma cells requires the interaction of P37 and Annexin A2
Source: PLoS One. 2017 Oct 4;12(10):e0184578. doi: 10.1371/journal.pone.0184578 (PMC5627893; doi:10.1371/journal.pone.0184578)
Supplement: S2 Table — A list of all the secondary antibodies for western blotting and immunoflourscence staining we used in this study. (DOCX) [file pone.0184578.s002.docx]

S2 Table. The Information of Secondary Antibodies for Western blotting and Immunoflourscence staining

| Catalog number | Name | Provider | Dilutions |
| --- | --- | --- | --- |
| 7074 | Anti-rabbit IgG, HRP-linked Antibody | Cell Signaling Technology, Inc | Western blotting 1:1000 |
| 7076 | Anti-mouse IgG, HRP-linked Antibody | Cell Signaling Technology, Inc | Western blotting 1:1000 |
| A-21206 | Donkey anti-Rabbit IgG (H+L) Highly  Cross-Adsorbed Secondary Antibody, Alexa Fluor 488 | Invitrogen Antibodies | IF-IC 1:500 |
| A-31570 | Donkey anti-Mouse IgG (H+L) Highly Cross-Adsorbed Secondary Antibody, Alexa Fluor 555 | Invitrogen Antibodies | IF-IC 1:500 |
| A-31572 | Donkey anti-Rabbit IgG (H+L) Highly  Cross-Adsorbed Secondary Antibody, Alexa  Fluor 555 | Invitrogen Antibodies | IF-IC 1:500 |
