# Supplementary material for: Mycoplasma-associated multidrug resistance of hepatocarcinoma cells requires the interaction of P37 and Annexin A2
Source: PLoS One. 2017 Oct 4;12(10):e0184578. doi: 10.1371/journal.pone.0184578 (PMC5627893; doi:10.1371/journal.pone.0184578)
Supplement: S3 Table — A. P values, t values and degree of freedom in Fig 1A, the MTT analysis the cell viability of HCC97L/Hep3B treated with increasing concentrations of AZI/MXF, were analyzed using paired two-tailed student’s t-test. (DOCX) [file pone.0184578.s003.docx]

S3 Table. The Statistical data of paired two-tailed student’s *t*-test in Figure 1. A

| Cell line | Drug | Concentration | *t* , df | *P* value |
| --- | --- | --- | --- | --- |
| HCC97L | MXF | 3μg/mL | *t*=0.2156 df=2 | 0.8493 |
|  |  | 5μg/mL | *t*=7.168 df=2 | 0.0189 |
|  |  | 10μg/mL | *t*=4.867 df=2 | 0.0397 |
|  |  | 20μg/mL | *t*=18.80 df=2 | 0.0028 |
|  | AZI | 5μg/mL | *t*=0.3458 df=2 | 0.7625 |
|  |  | 25μg/mL | *t*=10.08 df=2 | 0.0097 |
|  |  | 50μg/mL | *t*=4.641 df=2 | 0.0434 |
|  |  | 100μg/mL | *t*=40.08 df=1 | 0.0159 |
| Hep3B | MXF | 1μg/mL | *t*=2.792 df=2 | 0.1079 |
|  |  | 3μg/mL | *t*=2.968 df=2 | 0.0972 |
|  |  | 5μg/mL | *t*=4.991 df=2 | 0.0379 |
|  |  | 10μg/mL | *t*=5.043 df=2 | 0.0371 |
|  | AZI | 5μg/mL | *t*=8.393 df=2 | 0.0139 |
|  |  | 25μg/mL | *t*=10.66 df=2 | 0.0087 |
|  |  | 50μg/mL | *t*=13.17 df=2 | 0.0057 |
|  |  | 100μg/mL | *t*=20.16 df=2 | 0.0025 |

df: degree of freedom
