# Supplementary material for: Mycoplasma-associated multidrug resistance of hepatocarcinoma cells requires the interaction of P37 and Annexin A2
Source: PLoS One. 2017 Oct 4;12(10):e0184578. doi: 10.1371/journal.pone.0184578 (PMC5627893; doi:10.1371/journal.pone.0184578)
Supplement: S4 Table — P values, t values and degree of freedom in Fig 1B, the qPCR analysis for the anti-mycoplasma effect of AZI/MXF treatment on HCC97L, Hep3B and PLC/PRF/5 cells, were analyzed using paired two-tailed student’s t-test. (DOCX) [file pone.0184578.s004.docx]

S4 Table. The Statistical data of paired two-tailed student’s *t*-test in Figure 1. B

| Cell line | Drug | Days of treatment | *t*, df | *P* value |
| --- | --- | --- | --- | --- |
| HCC97L | MXF | 3 | *t*=14490 df=2 | < 0.0001 |
|  |  | 5 | *t*=1116 df=2 | < 0.0001 |
|  |  | 7 | *t*=304.6 df=2 | < 0.0001 |
|  | AZI | 3 | *t*=61.36 df=2 | < 0.0001 |
|  |  | 5 | *t*=243.6 df=2 | < 0.0001 |
|  |  | 7 | *t*=66.46 df=2 | 0.0002 |
| Hep3B | MXF | 3 | *t*=4667 df=2 | < 0.0001 |
|  |  | 5 | *t*=115.9 df=2 | < 0.0001 |
|  |  | 7 | *t*=14.35 df=2 | 0.0048 |
|  | AZI | 3 | *t*=15.51 df=2 | 0.0041 |
|  |  | 5 | *t*=71.36 df=2 | 0.0002 |
|  |  | 7 | *t*=7.210 df=2 | 0.0187 |
| PLC/PRF/5 | MXF | 3 | *t*=0.7033 df=2 | 0.5547 |
|  |  | 5 | *t*=2.135 df=2 | 0.1663 |
|  |  | 7 | *t*=0.2057 df=2 | 0.8560 |
|  | AZI | 3 | *t*=0.7826 df=2 | 0.5158 |
|  |  | 5 | *t*=1.114 df=2 | 0.3813 |
|  |  | 7 | *t*=0.2724 df=2 | 0.8109 |

df: degree of freedom
