# Supplementary material for: Mycoplasma-associated multidrug resistance of hepatocarcinoma cells requires the interaction of P37 and Annexin A2
Source: PLoS One. 2017 Oct 4;12(10):e0184578. doi: 10.1371/journal.pone.0184578 (PMC5627893; doi:10.1371/journal.pone.0184578)
Supplement: S5 Table — IC50 values, F values, degrees of freedom (DFn, DFd) and P values of each curve in Fig 2, the MTT analysis for the cell viability of hepatocarcinoma cells treated with different chemotheraputic drugs alone or with the presence of anti-pcytoplasma antibiotics, were analyzed using extra-sum-of-squares F test. (DOCX) [file pone.0184578.s005.docx]

S5 Table. The Statistical data of extra-sum-of-squares *F* tests in Figure 2.

| Cell line | Block | Treatment | IC50 (μg/mL) | *F* | DFn, DFd | *P* value |
| --- | --- | --- | --- | --- | --- | --- |
| HCC97L | A | CDDP | 3.178 | 48.15 | 1,44 | ＜0.0001 |
|  |  | CDDP+MXF | 1.581 |  |  |  |
|  | B | CDDP | 3.178 | 41.94 | 1,44 | ＜0.0001 |
|  |  | CDDP+AZI | 2.087 |  |  |  |
| Hep3B | C | CDDP | 26.90 | 1.952 | 1,44 | ＞0.05 |
|  |  | CDDP+MXF | 36.04 |  |  |  |
|  | D | CDDP | 10.84 | 0.4206 | 1,44 | ＞0.05 |
|  |  | CDDP+AZI | 12.27 |  |  |  |
| PLC/PRF/5 | E | CDDP | 6.740 | 16.12 | 1,44 | ＞0.05 |
|  |  | CDDP+MXF | 4.979 |  |  |  |
|  | F | CDDP | 8.596 | 0.005746 | 1,44 | ＞0.05 |
|  |  | CDDP+AZI | 8.703 |  |  |  |
| HCC97L | G | GEM | 0.02616 | 95.15 | 1,44 | ＜0.0001 |
|  |  | GEM+MXF | 0.004620 |  |  |  |
|  | H | GEM | 0.03830 | 85.85 | 1,44 | ＜0.0001 |
|  |  | GEM+AZI | 0.004394 |  |  |  |
| Hep3B | I | GEM | 0.1909 | 86.78 | 1,44 | ＜0.0001 |
|  |  | GEM+MXF | 0.02929 |  |  |  |
|  | J | GEM | 0.2263 | 101.9 | 1,44 | ＜0.0001 |
|  |  | GEM+AZI | 0.01845 |  |  |  |
| PLC/PRF/5 | K | GEM | 1.466 | 3.615 | 1,44 | ＞0.05 |
|  |  | GEM+MXF | 0.7396 |  |  |  |
|  | L | GEM | 5.256 | 3.338 | 1,44 | ＞0.05 |
|  |  | GEM+AZI | 2.542 |  |  |  |
| HCC97L | M | MX | 0.03325 | 26.16 | 1,44 | ＜0.0001 |
|  |  | MX+MXF | 0.005937 |  |  |  |
|  | N | MX | 0.04069 | 50.92 | 1,44 | ＜0.0001 |
|  |  | MX+AZI | 0.003489 |  |  |  |
| Hep3B | O | MX | 0.03100 | 61.37 | 1,44 | ＜0.0001 |
|  |  | MX+MXF | 0.001648 |  |  |  |
|  | P | MX | 0.002188 | 40.78 | 1,44 | ＜0.0001 |
|  |  | MX+AZI | 0.0001300 |  |  |  |
| PLC/PRF/5 | Q | MX | 0.3277 | 5.137 | 1,44 | ＞0.05 |
|  |  | MX+MXF | 0.1676 |  |  |  |
|  | R | MX | 0.2916 | 3.802 | 1,44 | ＞0.05 |
|  |  | MX+AZI | 0.1580 |  |  |  |
| DFn: the degree of freedom for the numerator of the *F* ratio; DFd: the degree of freedom for the denominator of the *F* ratio | | | | | | |
