# Supplementary material for: Mycoplasma-associated multidrug resistance of hepatocarcinoma cells requires the interaction of P37 and Annexin A2
Source: PLoS One. 2017 Oct 4;12(10):e0184578. doi: 10.1371/journal.pone.0184578 (PMC5627893; doi:10.1371/journal.pone.0184578)
Supplement: S6 Table — IC50 values, F values, degrees of freedom (DFn, DFd) and P values of each curve in Fig 3A–3D, the MTT analysis for the effect of A2PP on the cell viability of HCC97L to chemotherapeutic drugs, were analyzed using extra-sum-of-squares F test. (DOCX) [file pone.0184578.s006.docx]

S6 Table. The Statistical data of extra-sum-of-squares *F* tests in Figure 3. A-D

| Block | Treatment | IC_50_ (μg/mL) | Compare Groups | *F* | DFn, DFd | *P* value |
| --- | --- | --- | --- | --- | --- | --- |
| A | GEM | 0.06273 | GEM vs. GEM+MXF | 188.7 | 1,44 | ＜0.0001 |
|  | GEM+MXF | 0.002694 | GEM vs. GEM+A2PP | 0.004364 | 1,44 | ＞0.05 |
|  | GEM+A2PP | 0.06361 | GEM+MXF vs. GEM+A2PP | 144.5 | 1,44 | ＜0.0001 |
| B | GEM | 0.06881 | GEM vs. GEM+MXF | 143.3 | 1,44 | ＜0.0001 |
|  | GEM+MXF | 0.004428 | GEM vs. GEM+A2PP | 43.66 | 1,44 | ＜0.0001 |
|  | GEM+A2PP | 0.01635 | GEM+MXF vs. GEM+A2PP | 23.21 | 1,44 | ＜0.0001 |
| C | GEM | 0.05127 | GEM vs. GEM+MXF | 97.44 | 1,44 | ＜0.0001 |
|  | GEM+MXF | 0.004148 | GEM vs. GEM+A2PP | 160.6 | 1,44 | ＜0.0001 |
|  | GEM+A2PP | 0.003291 | GEM+MXF vs. GEM+A2PP | 0.9006 | 1,44 | ＞0.05 |
| D | MX | 0.02771 | MXvs. MX+MXF | 5.685 | 1,44 | 0.0215 |
|  | MX+MXF | 0.008916 | MX vs. MX+A2PP | 11.69 | 1,44 | 0.0014 |
|  | MX+A2PP | 0.003930 | MX+MXF vs. MX+A2PP | 5.626 | 1,44 | 0.0221 |
| DFn: the degree of freedom for the numerator of the *F* ratio; DFd: the degree of freedom for the denominator of the *F* ratio | | | | | | |
