# Supplementary material for: Mycoplasma-associated multidrug resistance of hepatocarcinoma cells requires the interaction of P37 and Annexin A2
Source: PLoS One. 2017 Oct 4;12(10):e0184578. doi: 10.1371/journal.pone.0184578 (PMC5627893; doi:10.1371/journal.pone.0184578)
Supplement: S7 Table — P values, t values and degree of freedom in Fig 3E, MTT analysis for Cell viability of HCC97L cells treated with different concentrations of A2PP or vehicle DMSO, were analyzed using paired two-tailed student’s t-test. (DOCX) [file pone.0184578.s007.docx]

S7 Table. The Statistical data of paired two-tailed student’s *t-*test in Figure 3. E

| Treatment | *t*, df | *P* value |
| --- | --- | --- |
| A2PP-40μM | *t*=0.7898 df=2 | 0.5124 |
| A2PP-120μM | *t*=2.128 df=2 | 0.1671 |
| A2PP-160μM | *t*=0.8217 df=2 | 0.4976 |
| DMSO | *t*=0.3422 df=2 | 0.7648 |
| df: degree of freedom |  |  |
