# Supplementary material for: Mycoplasma-associated multidrug resistance of hepatocarcinoma cells requires the interaction of P37 and Annexin A2
Source: PLoS One. 2017 Oct 4;12(10):e0184578. doi: 10.1371/journal.pone.0184578 (PMC5627893; doi:10.1371/journal.pone.0184578)
Supplement: S8 Table — P values, t values and degree of freedom in Fig 4A, the intensity density analysis for western blotting, were analyzed using unpaired two-tailed student’s t-test. (DOCX) [file pone.0184578.s008.docx]

S8 Table. The Statistical data of unpaired two-tailed student’s *t*-test in Figure 4. A

| Protein | *t*, df | *P* value |
| --- | --- | --- |
| ABCB1 | *t*=0.07040 df=4 | 0.9473 |
| ABCG2 | *t*=0.9049 df=4 | 0.4167 |
| ABCC1 | *t*=1.840 df=4 | 0.1396 |
| df: degree of freedom | | |
